# Gene expression analysis in recurrent benign paroxysmal positional vertigo: a preliminary study

**DOI:** 10.3389/fneur.2023.1223996

**Published:** 2023-07-05

**Authors:** Eun Hye Oh, Jin-Ok Lee, Hyun Sung Kim, Ji-Yun Park, Seo Young Choi, Kwang-Dong Choi, Ji-Soo Kim, Jae-Hwan Choi

**Affiliations:** ^1^Department of Neurology, Pusan National University School of Medicine, Research Institute for Convergence of Biomedical Science and Technology, Pusan National University Yangsan Hospital, Yangsan, Republic of Korea; ^2^Department of Health Science and Technology, Graduate School of Convergence Science and Technology, Seoul National University, Seoul, Republic of Korea; ^3^Department of Neurology, Gyeongsang National University School of Medicine, Gyeongsang National University Changwon Hospital, Changwon, Republic of Korea; ^4^Department of Neurology, Ulsan University Hospital, University of Ulsan College of Medicine, Ulsan, Republic of Korea; ^5^Department of Neurology, Pusan National University Hospital, Pusan National University School of Medicine, Biomedical Research Institute, Busan, Republic of Korea; ^6^Dizziness Center, Clinical Neuroscience Center, Department of Neurology, Seoul National University Bundang Hospital, Seongnam, Republic of Korea

**Keywords:** benign paroxysmal positional vertigo (BPPV), gene expression profiling (GEP), bioinformatics analysis, oxidative stress, immune system

## Abstract

**Objectives:**

This study aimed to determine the pathophysiology of recurrent benign paroxysmal positional vertigo (BPPV) in young patients using gene expression profiling combined with bioinformatics analysis.

**Methods:**

Total RNA was extracted from the whole blood of four young patients with recurrent BPPV and four controls. The differentially expressed genes (DEGs) between the groups were screened using a microarray analysis based on the cutoff criteria of |log_2_ fold change| > 1 and an adjusted *p*-value of < 0.05. Functional enrichment analysis of DEGs was performed using Gene Ontology analysis, and the protein–protein interaction (PPI) network was constructed using the Search Tool for the Retrieval of the Interacting Genes database.

**Results:**

A total of 39 DEGs were detected between the BPPV and control samples, comprising 33 upregulated DEGs and six downregulated DEGs in the BPPV group. Functional enrichment analysis indicated that the upregulated DEGs were significantly enriched in terms related to metabolic processes and the immune system. Two main pathways were extracted from the PPI network: one was associated with oxidative phosphorylation and stress and the other with the adaptive immune system and extracellular matrix degradation.

**Conclusion:**

The findings of our bioinformatics analysis indicated that oxidative stress or extracellular matrix degradation due to immune-mediated inflammatory responses may contribute to the development of recurrent BPPV in young patients.

## Introduction

Benign paroxysmal positional vertigo (BPPV) is the most common cause of recurrent vertigo, with a lifetime prevalence of 2.4% (1). It is characterized by a brief spinning sensation and nystagmus triggered by a change in head position with respect to gravity (1). BPPV is attributed to otoconia dislodged from the utricle and displaced into the semicircular canals (1). The cause of BPPV is mostly unknown although there are various predisposing conditions that may damage and dislocate otoconia, such as head trauma, prolonged recumbent position, and labyrinthitis (1). Recurrences of BPPV are frequent with an annual recurrence rate of 15–20% (1, 2). Advanced age, migraine, and cardiovascular comorbidities had an increased risk of BPPV recurrence in the systematic review (3). Recent studies found that impaired calcium metabolism and vitamin D deficiency were associated with frequent BPPV recurrences (4, 5). However, these factors do not completely explain the pathogenesis of recurrent BPPV, especially in young patients.

Gene expression profiling of the whole transcriptome is increasingly adopted to explore disease-related genes, which provide valuable clues for pathophysiology. This study aimed to provide novel insights into the pathophysiology of recurrent BPPV using gene expression profiling combined with bioinformatics analysis.

## Materials and methods

Four patients with a history of recurrent BPPV and four healthy controls were included in this study. Detailed demographic and clinical characteristics of the patients are described in Table 1. The patients were all young women with an age of 37.8 ± 5.1 years (mean ± SD). The number of BPPV attacks ranged from three to eight, and the involved canals varied among the patients. They all had a normal vitamin D level and no history of migraine. The controls were women and volunteers without a history of BPPV.

**Table 1:** Demographic and clinical characteristics of the four participants.

**Patient no**	**Sex/age**	**Number of recurrences**	**Time elapsed since the first episode**	**The time interval between recurrences**	**Involved canal**
1	F/36	8	37 months	1–6 months	R PC and HC
2	F/37	3	12 months	3–5 months	R PC
3	F/33	6	22 months	3–4 months	L PC and HC
4	F/45	3	10 months	3–4 months	L AC and R PC

### RNA extraction and microarray analysis

Total RNA was extracted from the whole blood of four patients with recurrent BPPV and four healthy controls using the PAXgene Blood RNA Kit, as described previously (6). The purity and quantity of RNA were measured using a spectrophotometer (NanoDrop ND-1000, Thermo Fisher Scientific, Wilmington, DE, USA), and the quality was further measured using a bioanalyzer (Agilent 2100, Agilent Technologies) with an RNA Integrity Number.

Isolated total RNA was amplified, labeled, and hybridized using the Affymetrix Human Clariom S Assay according to the manufacturer's protocol. In brief, the purified RNA sample was transcribed to double-strand cDNA using the GeneChip Whole Transcript (WT) amplification kit. The cDNA was then fragmented and biotin-labeled with TdT (terminal deoxynucleotidyl transferase) using the GeneChip WT terminal labeling kit. Approximately 5.5 μg of the labeled cDNA was hybridized onto the Affymetrix GeneChip Array at 45°C for 16 h. After washing, the arrays were stained on the GeneChip Fluidics Station 450 and scanned using the GeneChip Scanner 3000. The raw data were analyzed using the Affymetrix GeneChip Command Console software.

### Identification of differentially expressed genes and functional enrichment analysis

The raw data were summarized and normalized using the Robust Multi-array Average module implemented in Affymetrix Power Tools. The differentially expressed genes (DEGs) between BPPV and control samples were screened based on fold change filtering combined with Student's *t*-tests. The false discovery rate (FDR) was controlled by the adjusted *p*-value using the Benjamini–Hochberg algorithm. Genes were considered DEGs when they conformed to the criteria of |log_2_ fold change| > 1 and an adjusted *p*-value of < 0.05.

To explain functional annotations for DEGs, Gene Ontology (GO) enrichment analysis was performed using g:Profiler (https://biit.cs.ut.ee/gprofiler/). GO is a type of bioinformatics analysis that provides current scientific knowledge about the functions of genes and their products and covers three biological terms: biological process (BP), cellular component (CC), and molecular function (MF). An adjusted *p*-value (FDR) of < 0.05 was used as the criterion for statistical significance.

### Construction and analysis of the protein–protein interaction network

DEGs were imported into the Search Tool for the Retrieval of Interacting Genes (STRING) online database (http://string-db.org) to obtain the protein–protein interaction (PPI) network. Pairs with a confidence score of ≥0.4 were selected, and the maximum number of interactors was set at 10. The PPI network was then constructed and visualized using Cytoscape software (version 2.8; www.cytoscape.org). The hub genes within the PPI network were extracted by calculating the following four topological properties using the Python NetworkX package: the degree (number of interactions per node), closeness (reciprocal of the sum of the geodesic distances from one node to each other node), betweenness (number of shortest paths that pass through each node), and subgraph centrality (weighted sum of all closed walks originating from each node). For each hub gene, the SuperPath, the unified pathway extracted from the different sources including KEGG, Cell Signaling Technology, R&D Systems, GeneGo, Reactome, BioSystems, Tocris Bioscience, and QIAGEN, was analyzed from GeneCards (http://genecards.org).

## Results

### DEG identification by microarray analysis

Based on the cutoff criteria (|log_2_ fold change| > 1 and an adjusted *p*-value of < 0.05), a total of 39 DEGs were detected between the BPPV and control samples, comprising 33 upregulated DEGs and six downregulated DEGs in the BPPV group (Supplementary Figure 1A and Supplementary Table 1). A hierarchical cluster analysis of the upregulated and downregulated DEGs revealed distinctive expression patterns between the two groups (Supplementary Figure 1B).

### Functional enrichment analysis of DEGs

**Figure 1:**
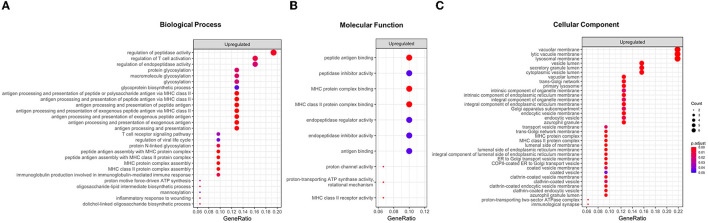
Results of a Gene Ontology (GO) enrichment analysis of upregulated differentially expressed genes (DEGs) in the BPPV group. All enriched GO terms for the biological process **(A)**, molecular function **(B)**, and cellular component **(C)**. The enriched terms are ranked according to their GeneRatio values (intersection size: the number of unique DEGs associated with GO terms). The adjusted *p*-values are labeled in different colors.

GO enrichment analysis revealed that the upregulated DEGs were enriched in 72 GO terms (27 BP, 35 CC, and 10 MF; Figure 1 and Supplementary Table 2). Remarkably, both BP and MF terms were divided into two categories such as metabolic processes and immune system (Figures 1A, B). The terms related to metabolic processes included the regulation of peptidase activity (GO:0052547), regulation of endopeptidase activity (GO:0052548), protein glycosylation (GO:0006486), macromolecule glycosylation (GO:0043413), glycoprotein biosynthetic process (GO:0009101), proton motive force-driven ATP synthesis in BP and endopeptidase inhibitor activity (GO:0004866), peptidase inhibitor activity (GO:0030414), endopeptidase regulator activity (GO:0061135), and proton channel activity (GO:0015252) in MF. Of the 33 upregulated DEGs, 12 were enriched in terms associated with metabolic processes: *PSMF1, PI3, GRN, PRELID1, CTSD, TIMP1, ALG3, DPM2, KRTCAP2, MPDU1, ATP6VOC*, and *ATP5FID* (Supplementary Table 2). Meanwhile, the terms linked with the immune system comprised regulation of T-cell activation (GO:0050863), antigen processing and presentation (GO:0019886, GO:0002495, GO:0002504, GO:0002478, GO:0019884, GO:0048002, and GO:0019882), MHC protein complex assembly (GO:0002396), immunoglobulin production involved in immunoglobulin-mediated immune response (GO:0002381) in BP, and MHC protein complex binding (GO:0023023), antigen binding (GO:0003823), and MHC class II receptor activity (GO:0032395) in MF. These terms consisted of six upregulated DEGs: *LAT, PRELID1, CTSD, HLA-DPB1, HLA-DQB1*, and *HLA-DRB1* (Supplementary Table 2). Regarding CC, the upregulated DEGs were mostly enriched in terms of intracellular anatomical structure, including lysosomal membrane (GO:0005765), lytic vacuole membrane (GO:0098852), and secretory granule lumen (GO:0034884; Figure 1C). The downregulated DEGs were enriched in 33 BP terms (Supplementary Table 2). However, since only one DEG was associated with each term, their significance seemed to be low compared with those of upregulated DEGs.

### PPI network construction and hub gene analysis

**Figure 2:**
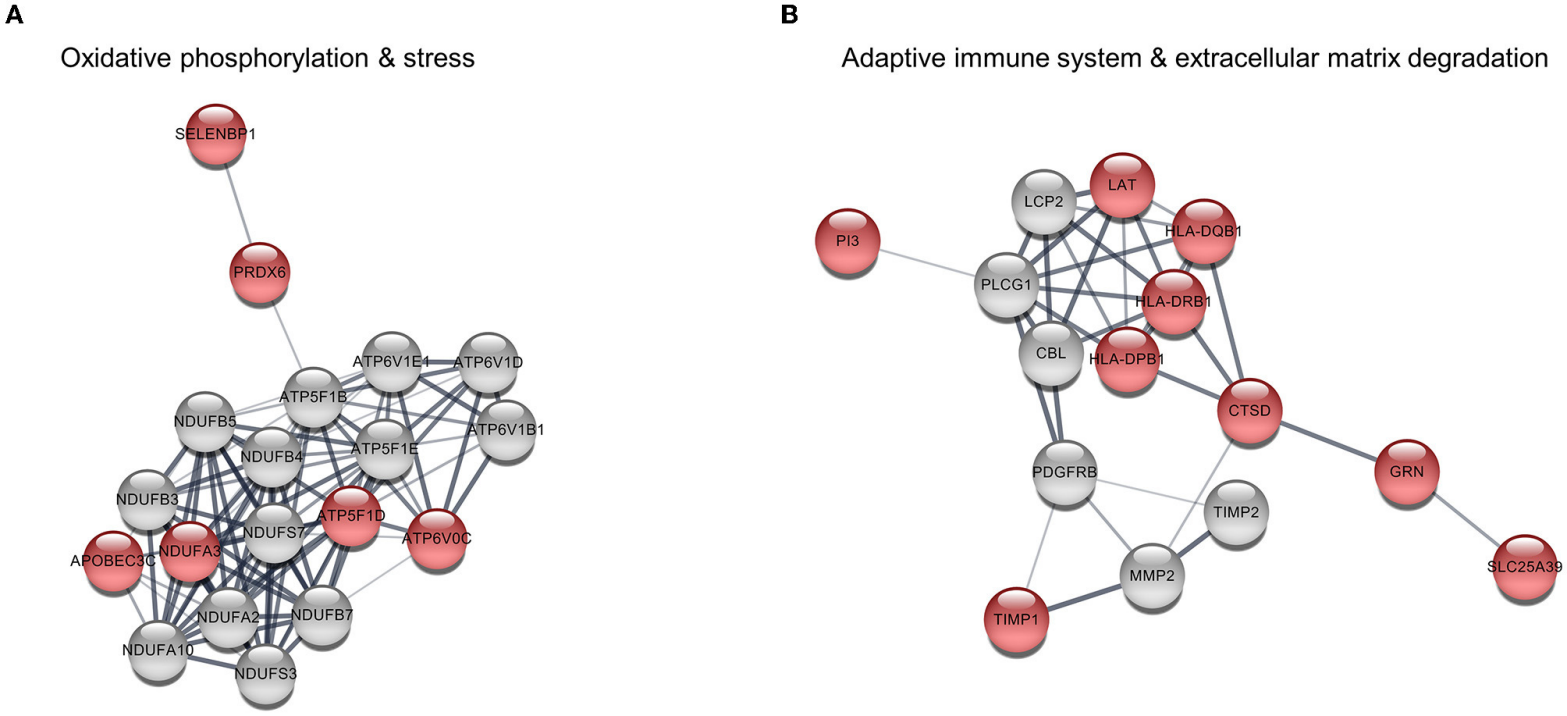
Protein–protein interaction (PPI) among upregulated differentially expressed genes (DEGs). **(A)** Pathway 1 is associated with oxidative phosphorylation and stress and consists of six upregulated DEGs (red nodes) and 13 interacting genes (gray nodes). **(B)** Pathway 2 is related to the adaptive immune system and extracellular matrix degradation comprising nine upregulating DEGs and six interacting genes.

Since the upregulated DEGs were significantly enriched in terms of metabolic processes and the immune system in the GO enrichment analysis, their PPI network was constructed using the STRING database. The PPI network contained 268 edges and 53 nodes, including 22 upregulated DEGs. It was notable that the PPI network was divided into two main pathways: one was closely associated with oxidative phosphorylation and stress in which six upregulated DEGs participated, while the other was related to the adaptive immune system and extracellular matrix degradation which included nine upregulating DEGs (Figure 2 and Table 2). The hub genes in the networks are listed in Supplementary Table 3. Among them, five upregulated DEGs were included in the top ten hub genes of each topological property: *ATP5F1D, NDUFA3, PRDX6, HLA-DRB1*, and *CTSD*.

**Table 2:** Upregulated differentially expressed genes (DEGs) associated with two main pathways.

**Gene symbol**	**Gene name**	**Log_2_ fold change**	**Adjusted *p*-value**
**Pathway 1: oxidative phosphorylation and stress**			
*ATP5F1D*	ATP synthase F1 and subunit delta	1.504842	0.016072
*APOBEC3C*	Apolipoprotein B m RNA-editing enzyme and catalytic polypeptide-kike 3C	1.397134	0.006893
*SELENBP1*	Selenium-binding protein 1	1.311559	0.045333
*PRDX6*	Peroxiredoxin 6	1.162456	0.023388
*NDUFA3*	NADH-ubiquinone oxidoreductase subunit A3	1.101548	0.010271
*ATP6V0C*	ATPase, H+ transporting, lysosomal, 16-KD, and V0 subunit C	1.071995	0.006893
**Pathway 2: adaptive immune system and extracellular matrix degradation**			
*PI3*	Proteinase inhibitor 3	1.791839	0.014859
*HLA-DPB1*	Major histocompatibility complex, class II, and DP beta-1	1.238880	0.015931
*HLA-DRB1*	Major histocompatibility complex, class II, and DR beta-1	1.189955	0.031353
*SLC25A39*	Solute carrier family 25 and member 39	1.156636	0.016113
*TIMP1*	Tissue inhibitor of metalloproteinase 1	1.081967	0.027708
*CTSD*	Cathepsin D	1.037971	0.026302
*HLA-DQB1*	Major histocompatibility complex, class II, and DQ beta-1	1.037183	0.027708
*LAT*	Linker for activation of T cells	1.004836	0.012948
*GRN*	Granulin precursor	1.000852	0.019996

## Discussion

To the best of our knowledge, this is the first analysis of the global transcriptome of DEGs for young patients with recurrent BPPV. The preliminary results of this study indicated that upregulated DEGs in the BPPV group were mostly associated with metabolic processes and the immune system. When the PPI network of these upregulated DEGs was investigated, the DEGs were predicted to be involved in two pathways with several interacting genes: one with oxidative phosphorylation and stress and the other with the adaptive immune system and extracellular matrix degradation. These results may be used to infer the pathophysiology of recurrent BPPV in young patients.

Otoconia consist of calcium carbonate crystals and organic matrix proteins (7). Mature otoconia are in a dynamic turnover process that is easily influenced by various factors. It is well-known that otoconia degeneration increases with age, which causes an increased risk of BPPV recurrence in elderly patients (3). In addition, normal calcium metabolism is particularly important for mineralizing and maintaining otoconia. There is increasing evidence of an association between impaired calcium metabolism and BPPV developments (4). Oxidative stress is a phenomenon caused by an imbalance between the production and accumulation of reactive oxygen species (ROS) in the cells and tissues. Previous studies have investigated the direct role of oxidative stress in BPPV and found that oxidative homeostasis differed significantly between the BPPV and control groups (8–10). The association between oxidative stress and BPPV is not fully understood, but altered calcium metabolism by oxidative stress has been suggested as the pathophysiology of BPPV (8, 9). The present study identified several upregulated DEGs associated with oxidative phosphorylation in the BPPV group, including *ATP5F1D, APOBEC3C, SELENBP1, PRDX6, NDUFA3*, and *ATP6V0C*. These genes have many interactors in the PPI network, suggesting that altered oxidative phosphorylation leads to excessive ROS production. Oxidative stress may cause the endoplasmic reticulum—the main organelle for calcium storage—to increase the influx of calcium, which contributes to dysregulation of the environmental balance for otoconia development and maintenance (11). In addition, *ATP5F1D* and *ATP6V0C* exhibit proton channel activity that maintains the pH of the extracellular environment. Optimal pH regulation is also important in the normal development and maintenance of the otoconia in the inner ear (7).

Alternatively, given the detection of upregulated DEGs associated with the immune system in this study, an altered immune response could also be involved in BPPV pathophysiology. The inner ear is controlled by systemic T lymphocytes for immune responses because it has connections with the cervical lymph nodes and the ability to produce cytokines in the spiral ligament (12). Previous studies have revealed the role of immune-mediated inflammatory processes in BPPV, such as the elevation of the serum macrophage migration inhibitory factor or pro-inflammatory mediators (9, 13). A higher frequency of BPPV has also been found among patients with giant-cell arteritis and systemic sclerosis (14, 15). In this study, the upregulated DEGs related to the immune system were connected by the extracellular matrix organization pathway, suggesting that immune-mediated inflammatory processes affect the organization of otolith matrix proteins in BPPV. Among the proteins involved in otoconia biomineralization, the otoconin-90 is the main matrix protein and has the ability of calcium binding (7). The otolin-1, located in the extracellular matrix of the inner ear, is a scaffold protein for otoconia and is responsible for calcium trimerization and binding (7). There are also numerous otolith-anchoring proteins such as the otogelin, α-tectorin, and β-tectorin (7). The GO enrichment analysis performed in this study revealed that many upregulated DEGs were associated with metabolic processes such as peptidase activity, protein glycosylation, and glycoprotein biosynthetic process. Especially *CTSD* and *TIMP1*, which were involved in extracellular matrix degradation, were strongly connected with the immune system pathway in the PPI network analysis. The immune-mediated inflammatory response may therefore contribute to the displacement of otoconia from the utricle via otolith matrix protein degradation in BPPV.

This study has some potential limitations. Our results were based on a small sample, so the expression levels of the DEGs should be verified using quantitative reverse transcription PCR for a larger number of patients. Because the enrolled patients were young women with recurrent BPPV, the results from this study may not be generalized to elderly patients with BPPV or patients with a single episode of BPPV. Finally, we obtained gene expression profiling from the peripheral blood. This approach may not reflect the pathological state of the inner ear well. Thus, transcriptomic analysis using endolymph in the inner ear could be a better method for identifying the underlying pathogenesis of BPPV.

In conclusion, this preliminary study has identified the upregulated DEGs associated with the metabolic processes and the immune system in young patients with recurrent BPPV. The findings of our bioinformatics analysis indicated that these DEGs are likely to contribute to BPPV development through oxidative stress or extracellular matrix degradation. Further studies involving larger samples are required to verify the present preliminary results.

## Data availability statement

The gene expression data analyzed for this study can be found in the Gene Expression Omnibus under accession number GSE231890: https://www.ncbi.nlm.nih.gov/geo/query/acc.cgi?acc=GSE231890.

## Ethics statement

All experiments followed the tenets of the Declaration of Helsinki, and informed consents were obtained after the nature and possible consequences of this study had been explained to the participants. This study was approved by the institutional review boards of Pusan National University Yangsan Hospital (04-2020-029).

## Author contributions

EO and J-OL analyzed and interpreted the data and wrote the manuscript. HK, J-YP, SC, K-DC, and J-SK analyzed and interpreted the data. J-HC designed and conceptualized the study, interpreted the data, and revised the manuscript. All authors contributed to the article and approved the submitted version.
