# Supplementary material for: Gene expression analysis in recurrent benign paroxysmal positional vertigo: a preliminary study
Source: Front Neurol. 2023 Jul 5;14:1223996. doi: 10.3389/fneur.2023.1223996 (PMC10354243; doi:10.3389/fneur.2023.1223996)
Supplement: Supplementary file 1 [file Table_1.doc]

Supplementary Table 1. All differentially expressed genes

| Gene symbol | Transcript ID | Log_2_ fold change | Adjusted *p*-value |
| --- | --- | --- | --- |
| **Up-regulated** | | | |
| *PI3* | NM_002638 | 1.791839 | 0.014859 |
| *RNASE2* | NM_002934 | 1.660167 | 0.006893 |
| *ATP5F1D* | NM_001001975 | 1.504842 | 0.016072 |
| *PSMF1* | NM_006814 | 1.479249 | 0.006893 |
| *APOBEC3C* | NM_014508 | 1.397134 | 0.006893 |
| *SLC6A8* | NM_001142805 | 1.331572 | 0.015379 |
| *SELENBP1* | NM_001258288 | 1.311559 | 0.045333 |
| *ALG3* | NM_001006941 | 1.256636 | 0.010271 |
| *DPM2* | NM_003863 | 1.244676 | 0.016072 |
| *HLA-DPB1* | NM_002121 | 1.23888 | 0.015931 |
| *FAM117A* | NM_030802 | 1.197085 | 0.006893 |
| *HLA-DRB1* | NM_001243965 | 1.189955 | 0.031353 |
| *H2BC14* | NM_175055 | 1.189892 | 0.03684 |
| *PRDX6* | NM_004905 | 1.162456 | 0.023388 |
| *SLC25A39* | NM_001143780 | 1.156636 | 0.016113 |
| *MPDU1* | NM_004870 | 1.147351 | 0.020183 |
| *FHL3* | NM_001243878 | 1.120575 | 0.030234 |
| *TMEM109* | NM_024092 | 1.10924 | 0.016072 |
| *NDUFA3* | ENST00000303553 | 1.101548 | 0.010271 |
| *TIMP1* | NM_003254 | 1.081967 | 0.027708 |
| *ATP6V0C* | NM_001198569 | 1.071995 | 0.006893 |
| *WBP2* | NM_012478 | 1.070366 | 0.009248 |
| *BANF1* | NM_001143985 | 1.065818 | 0.0164 |
| *PRELID1* | NM_001271828 | 1.049932 | 0.024072 |
| *SHISA4* | NM_198149 | 1.047608 | 0.016072 |
| *CTSD* | NM_001909 | 1.037971 | 0.026302 |
| *HLA-DQB1* | NM_001243961 | 1.037183 | 0.027708 |
| *GLMP* | ENST00000480968 | 1.036556 | 0.020183 |
| *NRM* | NM_001270707 | 1.024436 | 0.006893 |
| *CCDC6* | NM_005436 | 1.023628 | 0.009492 |
| *LAT* | NM_001014987 | 1.004836 | 0.012948 |
| *GRN* | NM_002087 | 1.000852 | 0.019996 |
| *KRTCAP2* | NM_173852 | 1.000808 | 0.020183 |
| **Down-regulated** | | | |
| *PGRMC2* | NM_006320 | -1.14598845 | 0.006893 |
| *TMEM154* | NM_152680 | -1.10315327 | 0.006893 |
| *TMCC1* | NM_001017395 | -1.09779375 | 0.01693 |
| *RRM2B* | NM_001172477 | -1.06958327 | 0.014887 |
| *LYSMD2* | NM_001143917 | -1.01816093 | 0.019197 |
| *HNRNPA2B1* | NM_002137 | -1.00911125 | 0.010271 |

**Supplementary Table 2.** Gene Ontology (GO) enrichment analysis of differentially expressed genes (DEGs).

| Regulation | GO term | Specific term | Count | *p*-value | Down-regulated DEGs |
| --- | --- | --- | --- | --- | --- |
| Up | BP | GO:0052547-regulation of peptidase activity | 6 | 8.67e-05 | *PSMF1/PI3/GRN/PRELID1/CTSD/TIMP1* |
|  |  | GO:0050863-regulation of T cell activation | 5 | 0.000223 | *LAT/HLA-DPB1/PRELID1/HLA-DQB1/HLA-DRB1* |
|  |  | GO:0052548-regulation of endopeptidase activity | 5 | 0.00061 | *PSMF1/PI3/PRELID1/CTSD/TIMP1* |
|  |  | GO:0019886- antigen processing and presentation of exogenous peptide antigen via MHC class II | 4 | 1.61e-07 | *HLA-DPB1/CTSD/HLA-DQB1/HLA-DRB1* |
|  |  | GO:0002495-antigen processing and presentation of peptide antigen via MHC class II | 4 | 2.71e-07 | *HLA-DPB1/CTSD/HLA-DQB1/HLA-DRB1* |
|  |  | GO:0002504-antigen processing and presentation of peptide or polysaccharide antigen via MHC class II | 4 | 3.43e-07 | *HLA-DPB1/CTSD/HLA-DQB1/HLA-DRB1* |
|  |  | GO:0002478-antigen processing and presentation of exogenous peptide antigen | 4 | 4.29e-07 | *HLA-DPB1/CTSD/HLA-DQB1/HLA-DRB1* |
|  |  | GO:0019884-antigen processing and presentation of exogenous antigen | 4 | 1.03e-06 | *HLA-DPB1/CTSD/HLA-DQB1/HLA-DRB1* |
|  |  | GO:0048002-antigen processing and presentation of peptide antigen | 4 | 3.37e-06 | *HLA-DPB1/CTSD/HLA-DQB1/HLA-DRB1* |
|  |  | GO:0019882-antigen processing and presentation | 4 | 2.77e-05 | *HLA-DPB1/CTSD/HLA-DQB1/HLA-DRB1* |
|  |  | GO:0006486-protein glycosylation | 4 | 0.000488 | *ALG3/DPM2/KRTCAP2/MPDU1* |
|  |  | GO:0043413-macromolecule glycosylation | 4 | 0.000488 | *ALG3/DPM2/KRTCAP2/MPDU1* |
|  |  | GO:0070085-glycosylation | 4 | 0.000661 | *ALG3/DPM2/KRTCAP2/MPDU1* |
|  |  | GO:0009101-glycoprotein biosynthetic process | 4 | 0.001722 | *ALG3/DPM2/KRTCAP2/MPDU1* |
|  |  | GO:0002399-MHC class II protein complex assembly | 3 | 2.24e-06 | *HLA-DPB1/HLA-DQB1/HLA-DRB1* |
|  |  | GO:0002503-peptide antigen assembly with MHC class II protein complex | 3 | 2.24e-06 | *HLA-DPB1/HLA-DQB1/HLA-DRB1* |
|  |  | GO:0002396-MHC protein complex assembly | 3 | 4.54e-06 | *HLA-DPB1/HLA-DQB1/HLA-DRB1* |
|  |  | GO:0002501-peptide antigen assembly with MHC protein complex | 3 | 4.54e-06 | *HLA-DPB1/HLA-DQB1/HLA-DRB1* |
|  |  | GO:0006487-protein N-linked glycosylation | 3 | 0.000206 | *ALG3/KRTCAP2/MPDU1* |
|  |  | GO:0002381-immunoglobulin production involved in immunoglobulin-mediated immune response | 3 | 0.000234 | *HLA-DPB1/HLA-DQB1/HLA-DRB1* |
|  |  | GO:0050852-T cell receptor signaling pathway | 3 | 0.001179 | *HLA-DPB1/HLA-DQB1/HLA-DRB1* |
|  |  | GO:1903900-regulation of viral life cycle | 3 | 0.001528 | *APOBEC3C/BANF1/HLA-DRB1* |
|  |  | GO:0090594-inflammatory response to wounding | 2 | 0.000352 | *GRN/TIMP1* |
|  |  | GO:0006488-dolichol-linked oligosaccharide biosynthetic process | 2 | 0.000396 | *ALG3/MPDU1* |
|  |  | GO:0006490-oligosaccharide-lipid intermediate biosynthetic process | 2 | 0.000442 | *ALG3/MPDU1* |
|  |  | GO:0015986-proton motive force-driven ATP synthesis | 2 | 0.000834 | *ATP6V0C/ATP5F1D* |
|  |  | GO:0097502-mannosylation | 2 | 0.001513 | *ALG3/DPM2* |
|  | MF | GO:0023026-MHC class II protein complex binding | 3 | 0.001179 | *HLA-DPB1/HLA-DQB1/HLA-DRB1* |
|  |  | GO:0023023-MHC protein complex binding | 3 | 0.001179 | *HLA-DPB1/HLA-DQB1/HLA-DRB1* |
|  |  | GO:0042605-peptide antigen binding | 3 | 0.001179 | *HLA-DPB1/HLA-DQB1/HLA-DRB1* |
|  |  | GO:0003823-antigen binding | 3 | 0.045904 | *HLA-DPB1/HLA-DQB1/HLA-DRB1* |
|  |  | GO:0004866-endopeptidase inhibitor activity | 3 | 0.045904 | *PSMF1/PI3/TIMP1* |
|  |  | GO:0030414-peptidase inhibitor activity | 3 | 0.045904 | *PSMF1/PI3/TIMP1* |
|  |  | GO:0061135-endopeptidase regulator activity | 3 | 0.045904 | *PSMF1/PI3/TIMP1* |
|  |  | GO:0032395-MHC class II receptor activity | 3 | 0.003466 | *HLA-DQB1/HLA-DRB1* |
|  |  | GO:0046933-proton-transporting ATP synthase activity, rotational mechanism | 2 | 0.008321 | *ATP6V0C/ATP5F1D* |
|  |  | GO:0015252-proton channel activity | 2 | 0.013973 | *ATP6V0C/ATP5F1D* |
|  | CC | GO:0005765-lysosomal membrane | 7 | 0.000134 | *ATP6V0C/HLA-DPB1/GRN/GLMP/CTSD/HLA-DQB1/HLA-DRB1* |
|  |  | GO:0098852-lytic vacuole membrane | 7 | 0.000134 | *ATP6V0C/HLA-DPB1/GRN/GLMP/CTSD/HLA-DQB1/HLA-DRB1* |
|  |  | GO:0005774-vacuolar membrane | 7 | 0.000201 | *ATP6V0C/HLA-DPB1/GRN/GLMP/CTSD/HLA-DQB1/HLA-DRB1* |
|  |  | GO:0034774-secretory granule lumen | 5 | 0.001298 | *RNASE2/GRN/PRDX6/CTSD/TIMP1* |
|  |  | GO:0060205-cytoplasmic vesicle lumen | 5 | 0.001298 | *RNASE2/GRN/PRDX6/CTSD/TIMP1* |
|  |  | GO:0031983-vesicle lumen | 5 | 0.001298 | *RNASE2/GRN/PRDX6/CTSD/TIMP1* |
|  |  | GO:0005766-primary lysosome | 4 | 0.001298 | *RNASE2/ATP6V0C/GRN/PRDX6* |
|  |  | GO:0042582-azurophil granule | 4 | 0.001298 | *RNASE2/ATP6V0C/GRN/PRDX6* |
|  |  | GO:0030176-integral component of endoplasmic reticulum membrane | 4 | 0.001298 | *HLA-DPB1/DPM2/HLA-DQB1/HLA-DRB1* |
|  |  | GO:0031227-intrinsic component of endoplasmic reticulum membrane | 4 | 0.001298 | *HLA-DPB1/DPM2/HLA-DQB1/HLA-DRB1* |
|  |  | GO:0005775-vacuolar lumen | 4 | 0.001298 | *RNASE2/GRN/PRDX6/CTSD* |
|  |  | GO:0030666-endocytic vesicle membrane | 4 | 0.00176 | *ATP6V0C/HLA-DPB1/HLA-DQB1/HLA-DRB1* |
|  |  | GO:0005802-trans-Golgi network | 4 | 0.004033 | *HLA-DPB1/GRN/HLA-DQB1/HLA-DRB1* |
|  |  | GO:0030139-endocytic vesicle | 4 | 0.010632 | *ATP6V0C/HLA-DPB1/HLA-DQB1/HLA-DRB1* |
|  |  | GO:0031301-integral component of organelle membrane | 4 | 0.013663 | *HLA-DPB1/DPM2/HLA-DQB1/HLA-DRB1* |
|  |  | GO:0098791-Golgi apparatus subcompartment | 4 | 0.013663 | *HLA-DPB1/GRN/HLA-DQB1/HLA-DRB1* |
|  |  | GO:0031300-intrinsic component of organelle membrane | 4 | 0.016327 | *HLA-DPB1/DPM2/HLA-DQB1/HLA-DRB1* |
|  |  | GO:0042613-MHC class II protein complex | 3 | 0.000134 | *HLA-DPB1/HLA-DQB1/HLA-DRB1* |
|  |  | GO:0042611-MHC protein complex | 3 | 0.00022 | *HLA-DPB1/HLA-DQB1/HLA-DRB1* |
|  |  | GO:0071556-integral component of lumenal side of endoplasmic reticulum membrane | 3 | 0.000249 | *HLA-DPB1/HLA-DQB1/HLA-DRB1* |
|  |  | GO:0098553-lumenal side of endoplasmic reticulum membrane | 3 | 0.000249 | *HLA-DPB1/HLA-DQB1/HLA-DRB1* |
|  |  | GO:0098576-lumenal side of membrane | 3 | 0.000387 | *HLA-DPB1/HLA-DQB1/HLA-DRB1* |
|  |  | GO:0012507-ER to Golgi transport vesicle membrane | 3 | 0.001298 | *HLA-DPB1/HLA-DQB1/HLA-DRB1* |
|  |  | GO:0030669-clathrin-coated endocytic vesicle membrane | 3 | 0.001506 | *HLA-DPB1/HLA-DQB1/HLA-DRB1* |
|  |  | GO:0035578-azurophil granule lumen | 3 | 0.002574 | *RNASE2/GRN/PRDX6* |
|  |  | GO:0045334-clathrin-coated endocytic vesicle | 3 | 0.002574 | *HLA-DPB1/HLA-DQB1/HLA-DRB1* |
|  |  | GO:0030134-COPII-coated ER to Golgi transport vesicle | 3 | 0.002702 | *HLA-DPB1/HLA-DQB1/HLA-DRB1* |
|  |  | GO:0032588-trans-Golgi network membrane | 3 | 0.003098 | *HLA-DPB1/HLA-DQB1/HLA-DRB1* |
|  |  | GO:0030665-clathrin-coated vesicle membrane | 3 | 0.004023 | *HLA-DPB1/HLA-DQB1/HLA-DRB1* |
|  |  | GO:0030662-coated vesicle membrane | 3 | 0.012888 | *HLA-DPB1/HLA-DQB1/HLA-DRB1* |
|  |  | GO:0030136-clathrin-coated vesicle | 3 | 0.014443 | *HLA-DPB1/HLA-DQB1/HLA-DRB1* |
|  |  | GO:0030658-transport vesicle membrane | 3 | 0.016327 | *HLA-DPB1/HLA-DQB1/HLA-DRB1* |
|  |  | GO:0030135-coated vesicle | 3 | 0.041066 | *HLA-DPB1/HLA-DQB1/HLA-DRB1* |
|  |  | GO:0001772-immunological synapse | 2 | 0.010756 | *LAT/HLA-DRB1* |
|  |  | GO:0016469-proton-transporting two-sector ATPase complex | 2 | 0.012888 | *ATP6V0C/ATP5F1D* |
| Down | BP | GO:0015886-heme transport | 1 | 0.040393 | *PGRMC2* |
|  |  | GO:0031053-primary miRNA processing | 1 | 0.040393 | *HNRNPA2B1* |
|  |  | GO:0006264-mitochondrial DNA replication | 1 | 0.040393 | *RRM2B* |
|  |  | GO:1901678-iron coordination entity transport | 1 | 0.040393 | *PGRMC2* |
|  |  | GO:0070316-regulation of G0 to G1 transition | 1 | 0.040393 | *RRM2B* |
|  |  | GO:0009200-deoxyribonucleoside triphosphate metabolic process | 1 | 0.040393 | *RRM2B* |
|  |  | GO:0009263-deoxyribonucleotide biosynthetic process | 1 | 0.040393 | *RRM2B* |
|  |  | GO:0009265-2'-deoxyribonucleotide biosynthetic process | 1 | 0.040393 | *RRM2B* |
|  |  | GO:0046385-deoxyribose phosphate biosynthetic process | 1 | 0.040393 | *RRM2B* |
|  |  | GO:0045023-G0 to G1 transition | 1 | 0.040393 | *RRM2B* |
|  |  | GO:0048025-negative regulation of mRNA splicing, via spliceosome | 1 | 0.040393 | *HNRNPA2B1* |
|  |  | GO:0032042-mitochondrial DNA metabolic process | 1 | 0.040393 | *RRM2B* |
|  |  | GO:1902254-negative regulation of intrinsic apoptotic signaling pathway by p53 class mediator | 1 | 0.040393 | *RRM2B* |
|  |  | GO:0033119-negative regulation of RNA splicing | 1 | 0.040393 | *HNRNPA2B1* |
|  |  | GO:0010971-positive regulation of G2/M transition of mitotic cell cycle | 1 | 0.040393 | *RRM2B* |
|  |  | GO:0050686-negative regulation of mRNA processing | 1 | 0.040393 | *HNRNPA2B1* |
|  |  | GO:0000002-mitochondrial genome maintenance | 1 | 0.040393 | *RRM2B* |
|  |  | GO:1902751-positive regulation of cell cycle G2/M phase transition | 1 | 0.040393 | *RRM2B* |
|  |  | GO:1902253-regulation of intrinsic apoptotic signaling pathway by p53 class mediator | 1 | 0.040393 | *RRM2B* |
|  |  | GO:1901797-negative regulation of signal transduction by p53 class mediator | 1 | 0.040765 | *RRM2B* |
|  |  | GO:1904358-positive regulation of telomere maintenance via telomere lengthening | 1 | 0.041466 | *HNRNPA2B1* |
|  |  | GO:0009394-2'-deoxyribonucleotide metabolic process | 1 | 0.041466 | *RRM2B* |
|  |  | GO:0014075-response to amine | 1 | 0.041466 | *RRM2B* |
|  |  | GO:0009262-deoxyribonucleotide metabolic process | 1 | 0.041466 | *RRM2B* |
|  |  | GO:0019692-deoxyribose phosphate metabolic process | 1 | 0.041466 | *RRM2B* |
|  |  | GO:0060612-adipose tissue development | 1 | 0.041466 | *PGRMC2* |
|  |  | GO:0051972-regulation of telomerase activity | 1 | 0.041698 | *HNRNPA2B1* |
|  |  | GO:0032206-positive regulation of telomere maintenance | 1 | 0.042936 | *HNRNPA2B1* |
|  |  | GO:0035196-production of miRNAs involved in gene silencing by miRNA | 1 | 0.042936 | *HNRNPA2B1* |
|  |  | GO:0070918-primary sncRNA processing | 1 | 0.044683 | *HNRNPA2B1* |
|  |  | GO:0006826-iron ion transport | 1 | 0.044951 | *PGRMC2* |
|  |  | GO:1904356-regulation of telomere maintenance via telomere lengthening | 1 | 0.044951 | *HNRNPA2B1* |
|  |  | GO:0006406-mRNA export from nucleus | 1 | 0.044951 | *HNRNPA2B1* |

*BP* biological process, *CC* cellular component, *MF* molecular function

**Supplementary Table 3**. Top ten hub genes presented by four topological properties extracted from the protein-protein interaction network.

| Rank | Gene (value) | | | |
| --- | --- | --- | --- | --- |
|  | Degree | Closeness | Betweenness | Subgraph |
| 1 | ***ATP5F1D*** (0.341) | *ATP5F1B* (0.359) | *ATP5F1B* (0.047) | *ATP5F1E* (7111.39) |
| 2 | *ATP5F1B* (0.341) | ***ATP5F1D*** (0.344) | ***CTSD*** (0.038) | *NDUFB5* (7039.33) |
| 3 | *ATP5F1E* (0.341) | *ATP5F1E* (0.344) | *PLCG1* (0.027) | *NDUFS7* (6953.53) |
| 4 | *NDUFS7* (0.317) | *NDUFS7* (0.329) | *PDGFRB* (0.022) | ***ATP5F1D*** (6776.10) |
| 5 | *NDUFB5* (0.317) | *NDUFB5* (0.329) | ***PRDX6*** (0.021) | ***NDUFA3*** (6472.72) |
| 6 | ***NDUFA3*** (0.293) | ***NDUFA3*** (0.316) | ***GRN*** (0.016) | *NDUFA2* (6337.29) |
| 7 | *NDUFS3* (0.293) | *NDUFS3* (0.316) | *MMP2* (0.015) | *NDUFS3* (6337.29) |
| 8 | *NDUFA2* (0.293) | *NDUFA2* (0.316) | ***ATP5D*** (0.012) | *ATP5F1B* (6259.66) |
| 9 | *NDUFA10* (0.268) | *NDUFA10* (0.304) | ***HLA-DRB1*** (0.010) | *NDUFB7* (5406.77) |
| 10 | *NDUFB7* (0.268) | *NDUFB3* (0.293) | *ATP5F1E* (0.009) | *NDUFA10* (5296.74) |

The bold genes denote the up-regulated differentially expressed genes in the BPPV group.
