# Supplementary material for: Gene expression analysis in recurrent benign paroxysmal positional vertigo: a preliminary study
Source: Front Neurol. 2023 Jul 5;14:1223996. doi: 10.3389/fneur.2023.1223996 (PMC10354243; doi:10.3389/fneur.2023.1223996)
Supplement: Supplementary file 2 [file Data_Sheet_1.doc]

**SUPPLEMENTARY FIGURE LEGENDS**

(**A**) The volcano plot of differentially expressed genes (DEGs). Based on the cut-off criteria (|log_2_ fold change| > 1 and adjusted p-value < 0.05), a total of 39 DEGs are detected between the BPPV and control samples, comprising 33 up-regulated (red dots) and 6 down-regulated (blue dots) DEGs in the BPPV group. (**B**) The results of a hierarchical clustering analysis of up-regulated and down-regulated DEGs between the BPPV and control groups. Each group includes four individuals and each column represents one individual’s sample. The red and blue colors indicate high and low relative expression levels, respectively.
