# Supplementary figures and images for: Gene expression analysis in recurrent benign paroxysmal positional vertigo: a preliminary study
Source: Front Neurol. 2023 Jul 5;14:1223996. doi: 10.3389/fneur.2023.1223996 (PMC10354243; doi:10.3389/fneur.2023.1223996)

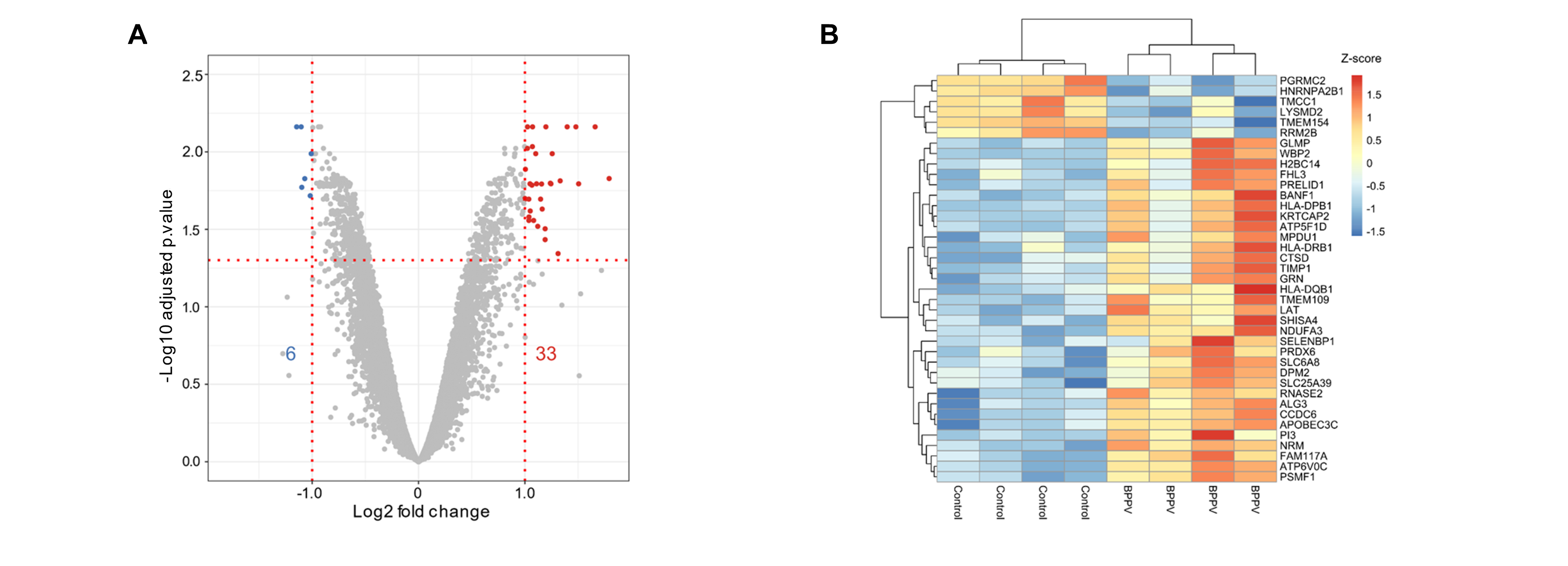

Supplement: Supplementary file 3 [file Image_1.TIF]
